# Alterations of regional homogeneity and functional connectivity in pituitary adenoma patients with visual impairment

**DOI:** 10.1038/s41598-017-13214-5

**Published:** 2017-10-12

**Authors:** Guidong Song, Jicheng Qiu, Chuzhong Li, Jiye Li, Songbai Gui, Haibo Zhu, Yazhuo Zhang

**Affiliations:** 1 Beijing Neurosurgical Institute, Capital Medical University, Beijing, 100050 China; 2 Department of Neurosurgery, Beijing Tiantan Hospital, Capital Medical University, Beijing, 100050 China; 3 Beijing Institute for Brain Disorders Brain Tumor Center, Beijing, 100050 China; 4 China National Clinical Research Center for Neurological Diseases, Beijing, 100050 China; 5 Sleep and Neuroimaging Center, Faculty of Psychology, Southwest University, Chongqing, 400715 China

## Abstract

Evidences have showed that the deprivation of vision can considerably alter the resting-state activity both within and beyond the visual cortices. However, the functional changes of the brain cortices related to partially vision-deprivation are still largely unknown. Using resting-state functional MR imaging, we quantitatively evaluated the regional homogeneity(ReHo) and functional connectivity(FC) changes between 25 pituitary adenoma patients with visual impairment and 25 healthy controls(HCs). Compared with HCs, PAs exhibited significant increased ReHo in the left superior occipital gyrus, bilateral middle occipital gyrus and reduced ReHo in the left inferior frontal gyrus and right middle temporal gyrus. PAs also showed decreased FC between vision-related area and higher-order cognitive brain areas. Furthermore, we identified that in the PAs group the FC between the left V1 and left V3 increased while the FC between left V2v and left V5 significantly decreased, the FC between left V4 area and the V3, V2d area increased. In our study, we identified that the ReHo and FC were altered between the vision-related cortices and other higher-order cognitive cortices along with disorganized functional connectivity within the visual system in PAs with visual impairment. These findings may provide important insights to understand the plasticity of visual network.

## Introduction

Primary sensory cortices and other brain regions can undergo functional and structural reorganizations due to the damage of a particular sensory modality, which are referred to as compensatory plasticity^1^. The visual system, which is one of the most complicated system in our brain, can dynamically remodel itself to adapt to the visual input changes through our life^2^. Visual input pass through the visual pathway and reach to the primary visual cortex, then the visual information is transferred to the higher-order visual cortices to process the information. The related brain areas associated with visual information constituting the complicated visual system^3,4^. The cortical process of the visual information is universally acknowledged going through two separated pathways, namely, the ventral and dorsal streams^5^. Visual deprivation provides a special model for investigating the complicated plastic mechanisms of the visual system. A great number of evidence showed the deprivation of vision can considerably alter the connectivity both within and beyond the visual cortices. The functional connectivity (FC) represents the temporal coherence of the blood oxygen level–dependent (BOLD) signals between separated brain areas. Previous studies found that compared with normal sighted HCs, the blind individuals exhibited decreased FC between the visual cortex and other cortices such as motor, somatosensory areas which were thought as non-visual areas^6^. Meanwhile, other studies found the FC between occipital cortex and prefrontal cortex, middle temporal/medial superior temporal areas significantly increased^7,8^.

Pituitary adenoma accounts for 15–20% of the intracranial neoplasms^9^, and the main chief complaints of the patients are visual acuity loss and visual field deficits due to the compression of the anterior visual pathway. The pituitary adenoma patients provide a unique model to study the cross-model vision-related changes of the brain due to partially visual deprivation of the adults. There are only few studies that focused on the functional changes of the brain cortices related to partially vision-deprivation caused by the compression of the anterior visual pathway. In a study, the researchers investigated the alterations of one pituitary adenoma patient before and after the chiasm decompression using functional magnetic resonance image(fMRI) techniques^10^. They found that the visual cortex of the affected eye activation area decreased compared with the normal side, and returned to normal after the surgical decompression of the chiasm. Another study explored the changes of brain resting-state activity related to vision in pituitary adenoma patients, their findings showed significant functional changes in the vision-related cortex of pituitary adenoma patients, within and beyond the visual cortices^11^. These studies only focused on the visual cortex and its subareas, the functional changes of the cortical visual processing pathway were not further investigated.

In our study, we recruited 25 pituitary adenomas with visual impairment and 25 normal controls, aiming not only to learn about the alterations of the visual cortex subareas but also the vision processing pathways and other higher cortices beyond visual cortex. We hope to explore the alterations of local synchronization and functional connectivity in pituitary adenoma patients(PAs) with visual impairment by using resting-state functional MR imaging(rs-fMRI).

## Results

### Demographic characteristics and visual profile

The demographic characteristics information are listed in Table 1. Two groups of participants showed no significant difference in age (*p* = 0.24), gender (*p* = 0.77), among two groups of participants. Twenty-five pituitary adenoma patients with visual impairments and 25 age- and sex matched HCs with normal vision were recruited. The detailed clinical and ophthalmological examinations results were recorded and summarized in Table 2. All of the patients demonstrated that the corrected vision acuity below 1.0 (20/20) or visual field defect more than 25% of the visual field at least one eye.

**Table 1:** Demographic characteristics of participants.

Characteristic	PAs(n = 25)	HC(n = 25)	***P***
Age (yr, mean ± std)	47.36 ± 11.97	43.76 ± 11.8	0.29
Gender (No.)			
Male	11(0.44)	14(0.56)	0.77
Female	12(0.48)	13(0.52)	

PAs = pituitary adenoma patients, HC = health controls.

**Table 2:** Summary of ophthalmological examinations results for PAs.

Patients	Sex	Age (year)	Duration (month)	Visual acuity		Visual field deficit(%)	
				LE	RE	LE	RE
PA1	F	39	8	1	1.2	75%	Normal
PA2	M	64	2	0.6	1.2	25%	Near-normal
PA3	M	32	24	0.6	0.8	20%	10%
PA4	F	35	12	0.03	1.5	Unable to detect	50%
PA5	M	41	12	0.8	0.6	10%	25%
PA6	F	53	6	0.6	0.6	50%	50%
PA7	M	66	24	0.5	0.8	NA	NA
PA8	F	50	12	0.1	0.8	75%	Near-normal
PA9	M	62	6	0.6	1	Near-normal	Near-normal
PA10	F	61	10	0.8	0.8	Near-normal	Near-normal
PA11	F	57	18	1	1.5	50%	50%
PA12	F	52	48	0.8	0.6	NA	NA
PA13	F	25	12	0.6	0.6	10%	50%
PA14	M	42	6	0.8	0.8	Near-normal	25%
PA15	M	49	6	0.5	1	25%	25%
PA16	F	31	10	0.6	0.8	Near-normal	Near-normal
PA17	F	47	6	0.25	0.25	50%	50%
PA18	M	52	4	Blind	1	Unable to detect	50%
PA19	M	59	2	0.15	0.1	35%	50%
PA20	F	34	12	1	1	Near-normal	Near-normal
PA21	M	62	2	0.8	0.8	Near-normal	Near-normal
PA22	M	57	3	0.6	0.5	Near-normal	20%
PA23	M	32	9	1	1.2	Near-normal	25%
PA24	M	43	12	0.5	0.6	25%	Near-normal
PA25	M	39	12	1	1	25%	Near-normal

PA = Pituitary Adenoma; M = Male; F = Female; LE = Left Eye; RE = Right Eye; NA = Not Available.

## Resting-state fMRI analysis

### ReHo analysis

**Figure 1:**
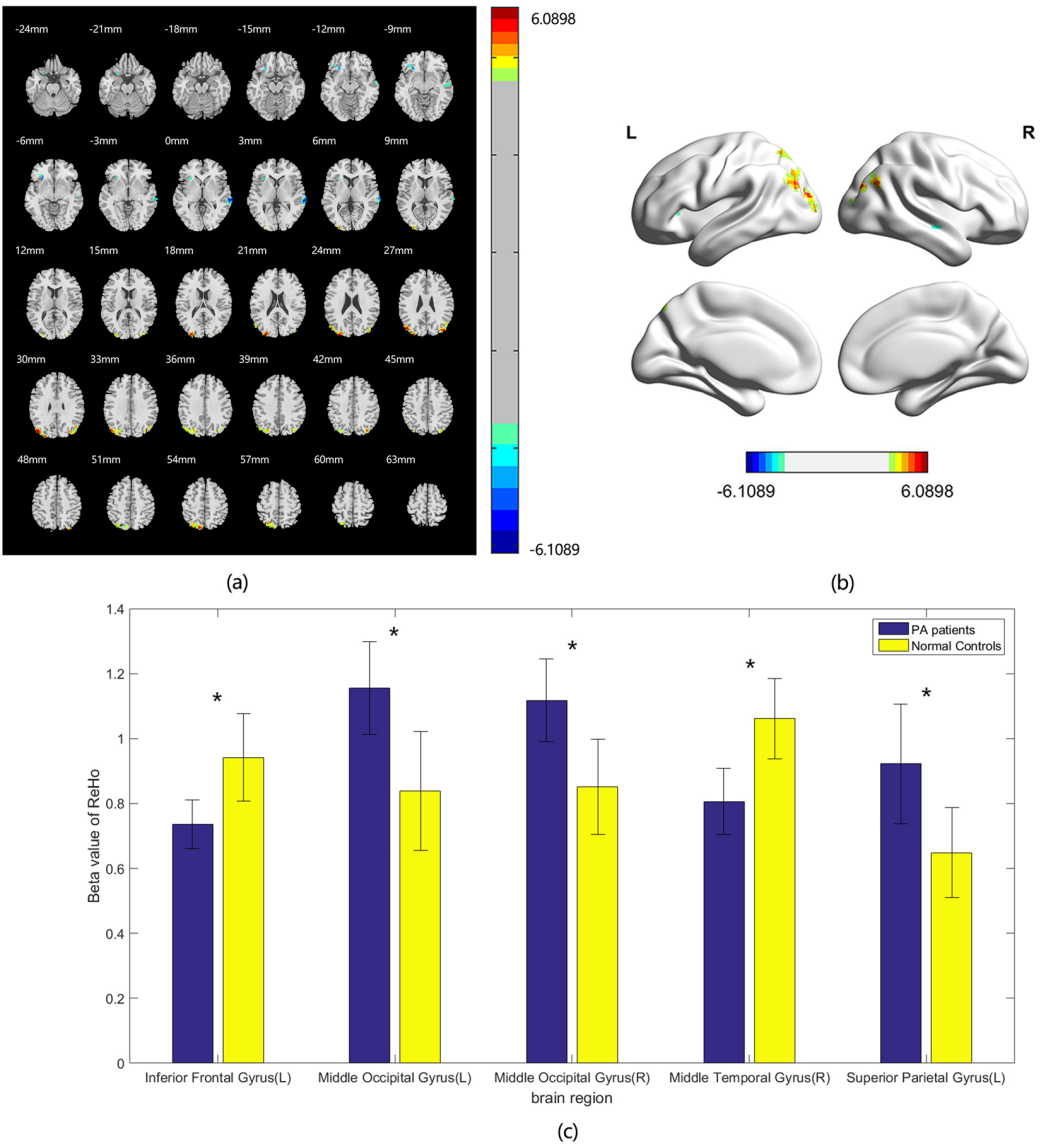
Group difference in ReHo between two groups. (**a**) Statistical parametric map (axial view). (**b**) Brain areas with significant differences are shown as projected onto a 3D brain model with the BrainNet viewer. (**c**) Brain regions with significant differences in beta values of ReHo. Compared to the normal controls, significantly increased ReHo in right middle temporal and right middle occipital gyrus are coloured in yellow-red, whereas significantly decreased ReHo in left inferior frontal gyrus, left middle occipital gyrus and left superior occipital gyrus are coloured in cyan-blue in PA patients. (*p* < 0.001 in voxel level, *p* < 0.05 in cluster level, GRF corrected).

Compared with HCs (*p* < 0.05 at the cluster level, GFR corrected), PAs group showed an significant increase of ReHo in the left superior occipital gyrus and bilateral middle occipital gyrus and a reduction of ReHo in the left inferior frontal gyrus and right middle temporal gyrus (Fig. 1a, Table 3). Brain areas with significant differences are projected onto a 3D brain model (Fig. 1b).

**Table 3:** Group difference in ReHo between two groups.

Brain region	Peak intensity	Peak MNI coordinate			Cluster size(voxels)
		x	y	z	
Inferior Frontal Gyrus(L)	5.5976	−27	27	−9	69
Middle Temporal Gyrus(R)	6.0444	66	−30	0	102
Middle Occipital Gyrus(L)	5.9609	−27	−90	21	279
Middle Occipital Gyrus(R)	5.4168	33	−81	48	116
Superior Occipital Gyrus (L)	5.9783	−12	−78	54	124

## FC analysis

### Voxel-wise FC analysis

**Figure 2:**
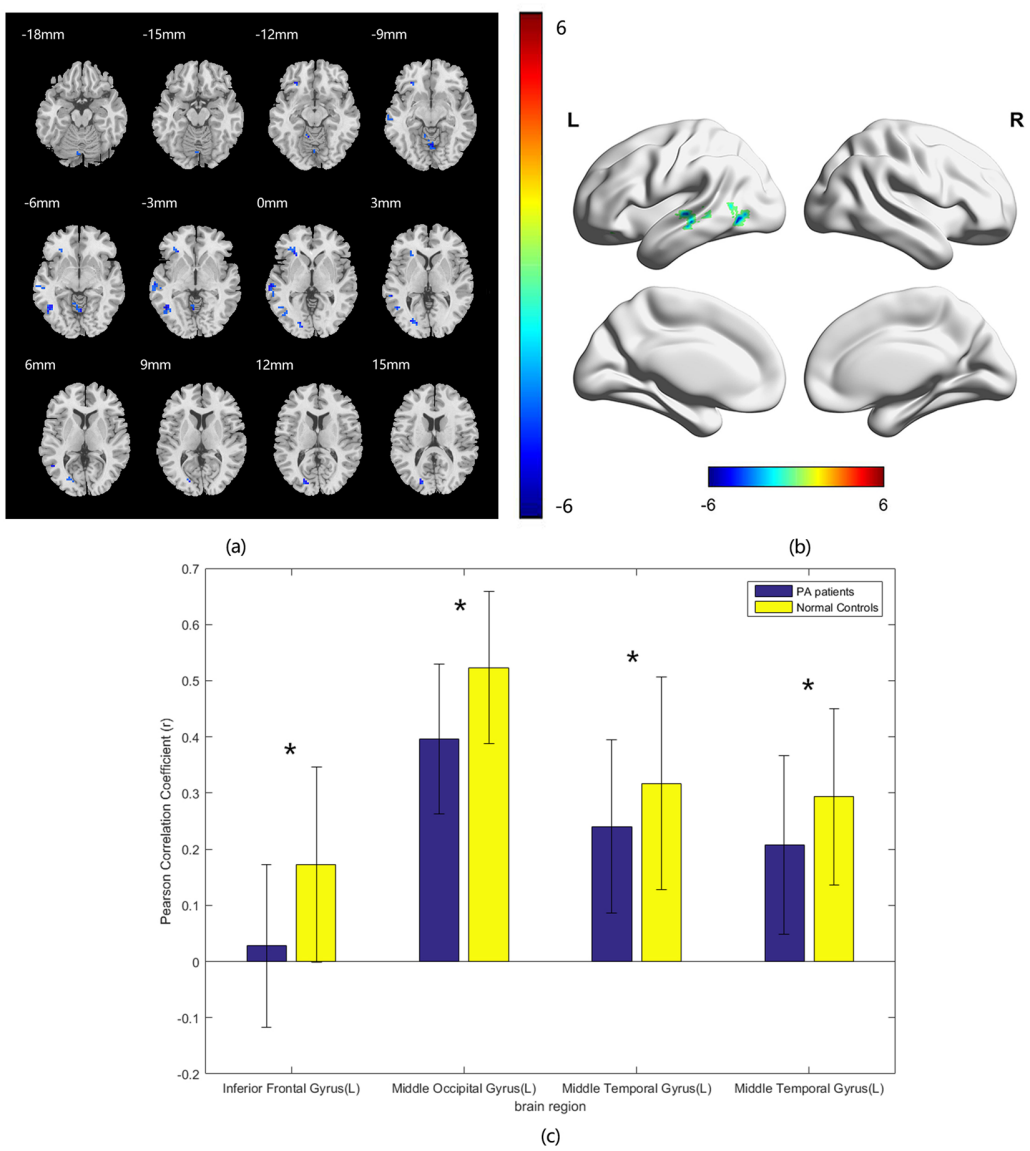
Group difference in voxel-wise FC between two groups. (**a**) Statistical parametric map (axial view). (**b**) Brain areas with significant differences are shown as projected onto a 3D brain model with the BrainNet viewer. (**c**) Brain areas with significant differences in Pearson correlation coefficients. For a seed placed in the BA 17 area, significantly decreased FC with left inferior frontal gyrus, left middle temporal gyrus, left middle temporal gyrus, left middle occipital gyrus, vermis and inferior cerebellum are coloured in cyan-blue in PA patients compared to the normal controls. (*p* < 0.001 in voxel level, *p* < 0.05 in cluster level, GRF corrected).

**Figure 3:**
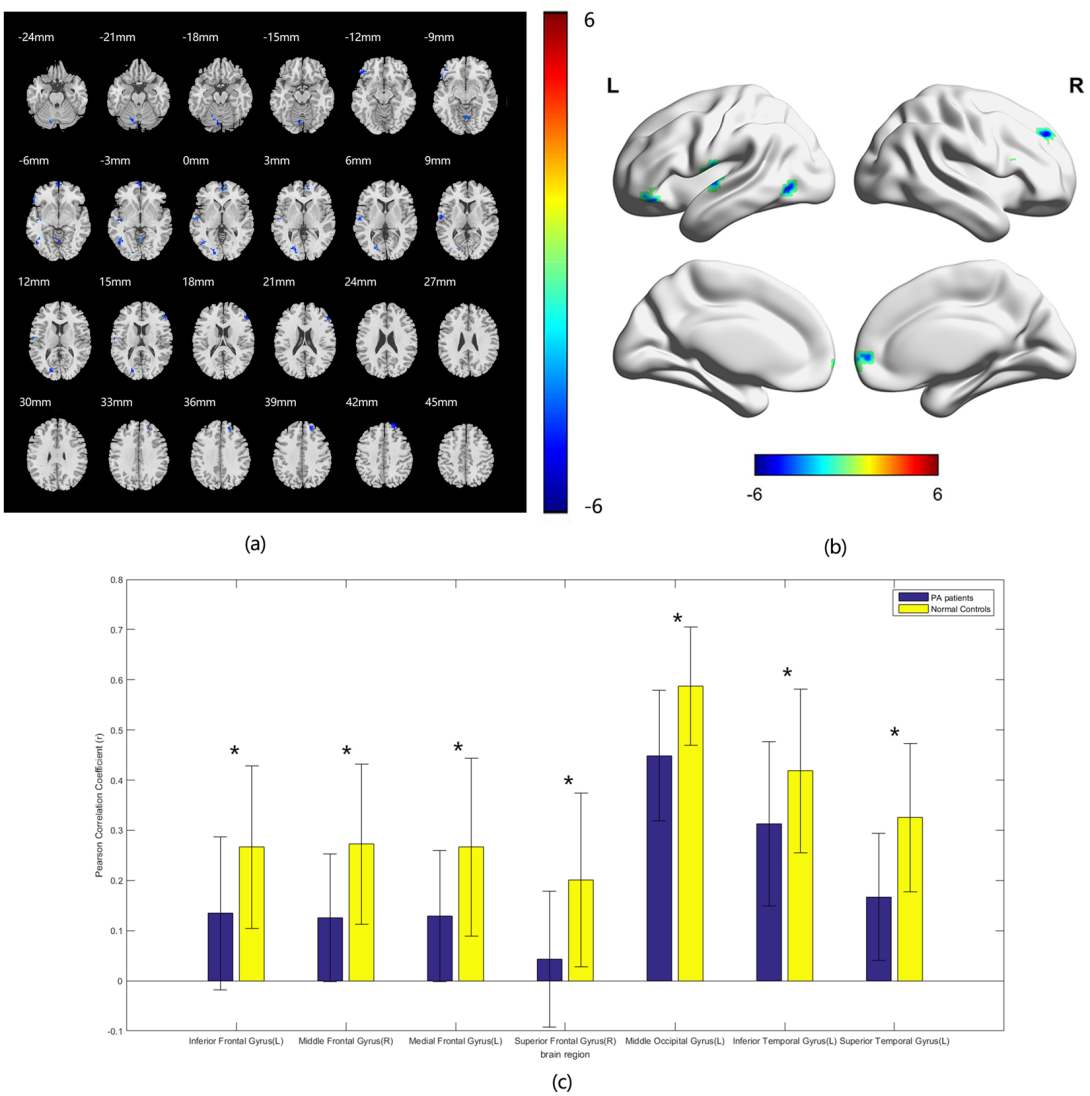
Group difference in FC between two groups. (**a**) Statistical parametric map (axial view). (**b**) Brain areas with significant differences are shown as projected onto a 3D brain model with the BrainNet viewer. (**c**) Brain areas with significant differences in Pearson correlation coefficients. For a seed placed in the BA 18 area, significantly decreased FC with left inferior frontal gyrus, left inferior temporal gyrus, left superior temporal gyrus, left middle occipital gyrus, left medial frontal gyrus, left middle frontal gyrus, superior frontal gyrus, inferior cerebellum, superior cerebellum and vermis are coloured in cyan-blue in PA patients compared to the normal controls. (*p* < 0.001 in voxel level, *p* < 0.05 in cluster level, GRF corrected).

The temporal correlations between visual cortices (V1, V2) and other brain regions are calculated by correlation analysis. The FC differences between the two groups of both sides of V1, V2 with other brain regions were assessed by voxel-wise two-sample t-test. In comparison with the HCs, the PA group showed decreased FC between bilateral the Brodmann area (BA) 17 area, left inferior frontal gyrus, left middle temporal gyrus, left middle temporal gyrus and left middle occipital gyrus (Fig. 2, Table 4). Compared with HCs, the PAs exhibited decreased FC between bilateral BA18 with bilateral superior temporal gyri, left inferior frontal gyrus, left inferior temporal gyrus, left middle occipital gyrus, left medial frontal gyrus and right middle frontal gyrus (Fig. 3, Table 5).

**Table 4:** Brain regions with BA17 FC differences between PAs compared with HCs.

Brain region	Peak intensity	Peak MNI coordinate			Cluster size(voxels)
		x	y	z	
Inferior Frontal Gyrus(L)	−4.4463	−27	33	−12	29
Middle Temporal Gyrus(L)	−4.603	−66	−27	0	36
Middle Temporal Gyrus(L)	−5.1074	−45	−57	−3	38
Middle Occipital Gyrus(L)	−4.7254	−21	−81	15	31
Vermis	−4.3389	3	−63	−6	36
Inferior Cerebellum(L)	−6.1615	−9	−45	−51	27

**Table 5:** Brain regions with BA18 FC differences between PAs compared with HCs.

Brain region	Peak intensity	Peak MNI coordinate			Cluster size(voxels)
		x	y	z	
Inferior Frontal Gyrus(L)	−5.142	−51	30	−12	26
Inferior Temporal Gyrus(L)	−4.7746	−48	−66	−6	27
Superior Temporal Gyrus(L)	−4.9173	−57	−12	6	43
Middle Occipital Gyrus(L)	−4.9216	−21	−81	15	41
Medial Frontal Gyrus(L)	−5.0881	0	63	−3	34
Middle Frontal Gyrus(R)	−4.7779	51	33	21	26
Superior Frontal Gyrus(R)	−4.9785	24	51	42	32
Inferior Cerebellum(L)	−6.1539	−9	−45	−51	28
Superior Cerebellum(L)	−5.1816	−6	−78	−18	32
Vermis	−4.3429	3	−63	−6	28

### ROI-wise FC analysis between subareas within visual network

**Figure 4:**
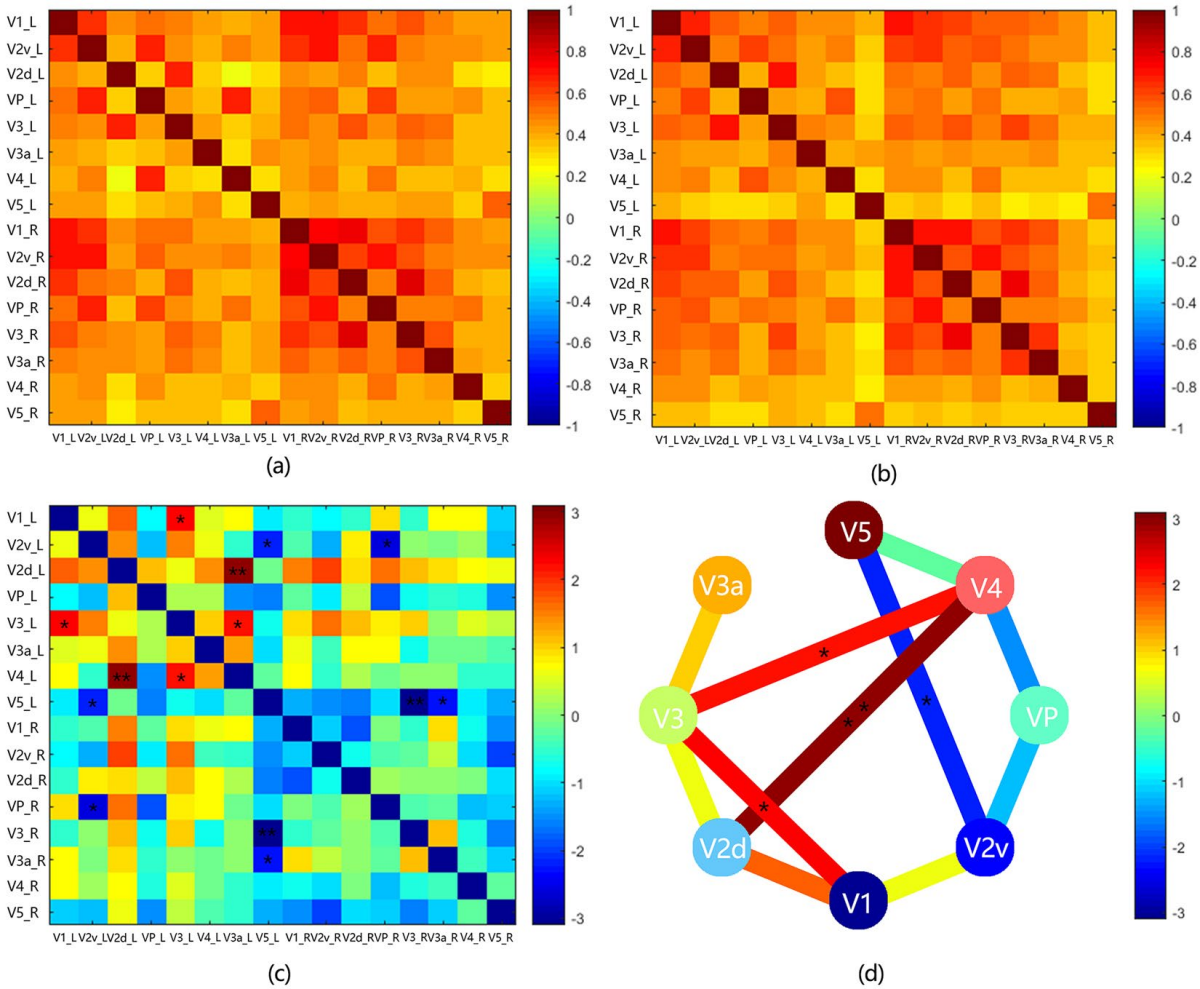
Group difference in ROI-wise FC between two groups. (**a**) Average FC (Pearson correlation coefficient) matrix between 16 nodes of normal controls. (**b**) Average FC matrix between 16 nodes of PA patients. (**c**) T value matrix of two sample *t*-test between Pearson correlation coefficients of two groups. (**d**)Visualization of *t* value matrix in the left hemisphere. Colours of the edges indicate the range of t value. (* means p < 0.05, ** means p < 0.01).

The synchrony of time series between the 16 nodes (8 nodes in each hemisphere) within visual network is measured by correlation analysis to determine the significant differences in FC among these nodes. Average FC of the 16 nodes is compared using two-sample *t*-test between Pearson correlation coefficients of two groups (Fig. 4). In the left hemisphere, compared with HCs the PAs showed increased FC between left V1 and left V3, decreased FC between left V2v and left V5. Also, in comparison with the normal controls, the FC in pituitary adenoma group significantly increased between the left V4 and V2d, V3 (Fig. 4c,d). Meanwhile, the PAs exhibited decreased FC between right VP with left V2v, and between left V5 and right V3, V3a (Fig. 4c).

## Discussion

In this study, we explored the regional and circuit visual-related resting-state activity alterations in pituitary adenomas patients with visual damages. As an emerging method, the resting-state fMRI is an efficient approach to exploring brain connectivity and plasticity with reliable results, particularly in patients who cannot perform task fMRI scans^12^.

It was hypothesized that the reorganization of the brain cortex occurs when the partially impaired vision failed to facilitate brain work efficiently in respond to the compromised visual input^13^. In our study, we found remarkable changes of the ReHo values in the visual areas and other cortices, compared with the HCs, the PAs group showed increased ReHo value in the left superior occipital gyrus and bilateral middle occipital gyri and and reduced ReHo value in the left inferior frontal gyrus and right middle temporal gyrus. Furthermore, we explored the functional connectivity patterns between the visual areas and other brain areas of the two groups. In comparison with the HCs, the PAs group presented decreased FC between visual areas and other brain regions (between bilateral V1 and left inferior frontal gyrus, left middle temporal gyrus, left middle temporal gyrus, left middle occipital gyrus and bilateral V2 with bilateral superior temporal gyri, left inferior frontal gyrus, left inferior temporal gyrus, left middle occipital gyrus, left medial frontal gyrus and right middle frontal gyrus). Finally, in comparison with the HCs, the pituitary adenoma subjects showed FC pattern alterations within the visual processing pathway.

The cortical visual system occupies the largest cortices of the brain, which is universally thought to be segregated into ventral stream and dorsal stream^14^. The ventral steam starts with visual area V1, relays to visual area V2, and finally reaches the inferior temporal cortex (IT cortex). It is also called “what stream”, which is associated with the object recognition. In contrast, the dorsal stream is referred to as “where pathway” that begins with visual area V1, passes through visual area V2, and visual area MT (middle temporal/V5), finally project dorsally to the parietal lobe. The function of the dorsal stream is associated with object locations^5^. In our study, we constructed the visual systems by selecting the key nodes of the visual stream to investigate the underlying changes caused by partial visual impairment.

ReHo represents the local spontaneous coherence of neural activity and is one of the most efficient, reliable, and widely used index to inspect the brain activity^15^. And the functional connectivity (FC) is widely used to characterize functional relationships in the spatially distant brain regions and has proven to be a very reliable method^16^. The alteration of ReHo or FC reflects an abnormal *changes of* brain activity^17^. In our study, vision-related areas (bilateral middle occipital gyri, left precuneus) showed a significantly increase of ReHo in the patients group. We also found the ReHo of the right middle temporal gyrus decreased significantly. There were several studies investigating the changes of the resting-state activity in partially visual impaired patients with glaucoma and other ophthalmological diseases^18–20^, these studies found there were significant changes in FC of the visual cortex between the visual cortex and other vision-related brain regions. And a study found that visual cortex could undergo structure and/or function changes following deprived retinal input^21^. Several studies have investigated the visual cortex changes in lesioned visual pathways^22,23^, the findings of which showed the recovery of the visual field perimetry correlates with the V1 BOLD signal changes in previously visually deprived areas. A study explored the alteration of vision-related resting-state activities in patients with anterior visual pathway lesions^11^. The results showed significant vision-related cortex subareas within the visual cortex FC and ReHo alterations. The above-mentioned studies indicated that when visual input to the visual cortex is damaged, the visual system may undergo compensatory changes. However, these researches mainly focus on the alteration of the vision-related cortex without studying the visual system itself. In our study, we did not find the ReHo changes in the primary visual cortex(V1), but the bilateral V2/V3 visual areas showed increased and the right middle temporal gyrus exhibited reduced ReHo. As we know, the intrinsic functional network is a robust system which can keep the brain function operating efficiently even under the circumstances of brain lesions. When the input of the visual cortex is compromised, the related visual areas can adjust their function to the alterations. Evidences showed that the primary visual cortex(V1), an early visual cortex, lacks of reorganization ability. However, the higher-order visual areas can make relevant changes to ensure the normal visual function^24,25^. Our results showed there were significant increased ReHo in bilateral middle occipital cortices(V2/V3), reflecting the dysfunction of the higher-order visual cortices.

A previous study investigated the multisensory plasticity of visual stream functions between congenitally blind people and sighted people using task fMRI^26^. Their results showed that compared with normal sighted controls, the congenitally blind participants exhibited activated extrastriate cortex and the auditory cortex. Furthermore, their findings showed that dorsal stream functions could develop through non-visual spatial information at an early age. These findings indicated that the visual deprivation may cause compensatory intra-modal and cross-modal plasticity in the visual system. A study investigated the microstructural differences between the dorsal and ventral visual pathways in congenitally blind, late blind and normal sighted controls. They revealed that fractional anisotropy in the ventral stream of congenital and late blind individuals decreased. It seems that the blindness can selectively affect the microstructure of the ventral visual stream^27^. Another study reported that in congenitally blind individuals, the functional connectivity in ventral stream areas is more easily to be affected than that in the dorsal stream^28^. These studies mainly explored the changes of visual streams of blind participants, but the changes of the visual streams associated with partial visual impairment are still largely unknown. In line with their studies, we identified that the FC between the left V1 and left V3 increased while the FC between left V2v and left V5 significantly decreased. The results indicated that the FC in ventral stream increased while the FC dorsal stream decreased. It seems that the increased FC in the ventral stream in pituitary adenoma subjects is a result of compensatory plasticity, affecting the intrinsic connectivity and functional coupling activities in patients with partial visual loss.

The current concepts of visual processing is that the visual system does not work separately. In fact, there are extensive cross-talks between the two streams^29^. Goodale *et al*. proposed three possible cross-stream talk models^30^, one of which is the ‘continuous cross-talk’ account. In this model, information can be transferred between the two streams at multiple stages in the processing pathways. In our study, the PAs group exhibited increased FC between left V4 area and the V3, V2d area. Our results indicated increased FC between ventral stream and dorsal stream, which may reflect the enhancement of functional integration process.

The BA17 areas is also called the primary visual cortices which receives the direct visual stimuli from the retina. A resting-state study found that primary visual cortex was functionally associated with other brain areas, including the precuneus, the precentral/postcentral gyrus, the middle frontal gyrus, the fusiform gyrus, the inferior/middle temporal gyrus. They indicated that the functional connectivity may be related with processes of memory-related mental imagery and/or visual memory consolidation^31^. In our study, we identified that the FC between the BA17 area and the left middle temporal gyrus, left middle temporal gyrus, left middle occipital gyrus diminished. The decreased FC between the BA17 area and these regions in pituitary adenoma patients may thus indicate the dysfunction of these processes. Jann *et al*. described in visual damaged patients the FC between the BA 17 area and language network decreased^32^. The FC decreased between the BA17 area and the left inferior frontal gyrus, which is crucial for the comprehension and production of language. This finding may reflect the dysfunction of visual and language information integration. Our study also demonstrated decreased FC between the primary visual cortex (BA17) and higher visual cortices (BA19), which indicated the visual information processing pathway from primary visual area to higher visual cortices may be compromised.

Our study showed decreased FC between the BA18 area and bilateral superior temporal gyri, left inferior frontal gyrus, left inferior temporal gyrus, left middle occipital gyrus, left medial frontal gyrus and right middle frontal gyrus in the PAs compared with the HCs. The FC decreased between the BA 18 area and the left inferior temporal gyrus, which plays a key function in visual, auditory and sensory integration as it will impair the integration of visual and other sensory stimuli. Also, the PAs group showed decreased FC between the BA 18 area and some higher-order cortices. The superior temporal gyrus is responsible for processing sounds and speech^33^.It was proved that the medial frontal gyrus may play a role in executive function^34^. Decreased FC between the BA 18 visual area and cognitive cortices may be caused by the decreased integration of visual information into these functions.

In our study, the FC between the BA17/18 area and the vermis, cerebellum decreased in PAs group. These findings may be a result of diminished input from the visual area, which may indicate the compromised integration of visual information and voluntary movement and postural balance.

## Conclusions

In our study, we identified that the ReHo and FC altered between the vision-related cortices and other higher-order cognitive cortices along with disorganized functional connectivity within the visual system in PA patients with visual impairment. These findings may provide important insights to understand the plasticity of visual network.

## Methods

### Participants

Twenty-five patients (aged 25–66, mean 47.36 ± 11.97 years old, including 11 females and 14 males) diagnosed with pituitary adenoma presented with visual impairment and 25 age, sex, and handedness-matched healthy controls (aged 23–64, mean 43.76 ± 11.48 years old, including 12 females and 13 males) with normal vision were recruited from Beijing Tiantan Hospital and the local community respectively. The patients inclusion criteria were: aged from 18–70 years old; no ophthalmologic diseases conformed by ophthalmologic examination and other intracranial lesions through MRI scan; visual impairment either with vision acuity below 1.0 (20/20)or visual field defect of more than 25% of the visual field at least one eye. For both the pituitary adenoma patients and health controls the visual acuity was assessed with the E chart. Using the standardized automated perimetry (Octopus900 Perimetry) to obtain the visual field data. All of the participants were fully informed prior to the study and signed consent forms. The study was granted ethical approval by the Institutional Review Board of Beijing Tiantan Hospital affiliated to Capital Medical University, and the study was carried out in accordance with the relevant guidelines and regulations.

### Image acquisition

The rs-fMRI data were obtained using a 3.0 Tesla Siemens scanner with a standard head coil. The rs-fMRI data were acquired with an echo-planar image sequence (30 axial slices, slice thickness/gap = 5/0.5 mm, repetition time = 2000 ms, echo time = 30 ms, acquisition matrix = 64 * 64, field of view(FOV) = 192 * 192 mm with an in-plane resolution of 3.0 * 3.0 mm). A T1 weighted sagittal anatomical image was also acquired (192 slices, slice thickness/gap = 1/0.5 mm, repetition time = 2530 ms, echo time = 2.55 ms, acquisition matrix = 512 * 512; flip angle = 12 deg, FOV = 256 * 256 mm with an in-plane resolution of 0.7 * 0.7 mm). All the participants were instructed to relax, stare at fixation point on the center screen and avoid thinking about anything.

### Data analysis

#### Data preprocessing

The rs-fMRI data were preprocessed using statistical Parametric Mapping toolbox (SPM12, http://www.fil.ion.ucl.ac.uk/spm) and Data Processing & Analysis of Brain Imaging toolbox (DPABI, http://www.restfmri.net/)^35^. The first 20 seconds images were removed to avoid transient signal changes. The remaining rs-fMRI data were firstly preprocessed by slice timing correction and head motion correction. Secondly, regression of nuisance covariates including mean white matter (WM), mean cerebrospinal fluid (CSF), linear trend and Friston’s 24 head motion regressors were performed with autoregressive models. Global signals were not regressed for it may negatively influence the results^36^. Thirdly, regressed data were spatially normalized to the Montreal Neurological Institute(MNI) space based on each subject’s own segmented T1-weighted image registration. Finally, spatial smoothness with a 4 * 4 * 4 mm full-width-half-maximum(FWHM) Gaussian kernel and band-pass filtering of each time series in 0.01–0.08 Hz were performed.

### Regional homogeneity(ReHo) analysis

As a data-driven method, ReHo was used to measure local synchronization of fluctuations of spontaneous blood oxygen level dependent(BOLD) signals with nearest neighbouring voxels^37^. We used DPABI to calculate each subject’s ReHo parametric map that each voxel presents the Kendall’s coefficient of concordance of its nearest neighbouring 27-voxels cluster based on unsmoothed preprocessed images. Two sample *t*-test of each voxel between patients and HCs on ReHo maps were performed after division by global mean value, Fisher z-transformation and spatial smoothness with a 4 * 4 * 4 mm full-width-half-maximum kernel. The gibbs random field (GRF) method was used to correct for multiple comparisons. The corrected value of *p* < 0.001 in voxel level and *p* < 0.05 in cluster level were used as the threshold.

### Seed-based resting state functional connectivity(FC) analysis

**Figure 5:**
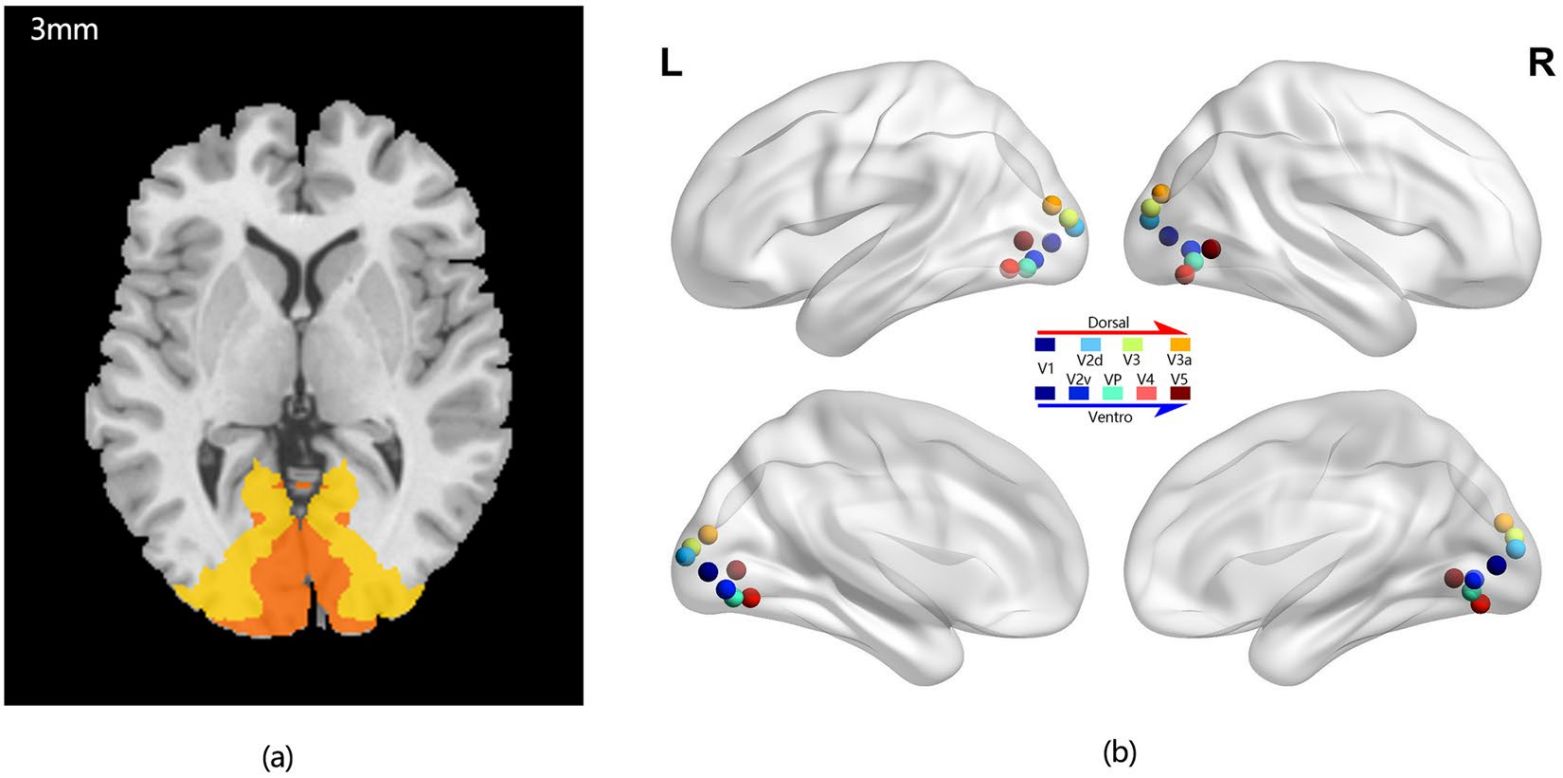
Definition of ROIs. (**a**) BA17(dark yellow) and BA18 (light yellow). (**b**) 16 nodes including bilateral V1, V2v,V2d, V3, V3a, VP, V4 and V5. (within 5-mm radius around coordinate).

As a priori-based method, seed-based FC analysis is one of the most direct ways to measure correlations between distant brain regions which includes voxel-wise and region-of-interest(ROI)-wise FC^38^. Higher-order visual processing pathway is considered to be divided into two different streams. The dorsal stream is considered as the “where pathway” and “how pathway” that associated with perception of the spatial locations of an object. The ventral stream is referred to as the “what pathway” which is responsible for object recognition and the storage of long-term memory. In this study, we defined these 16 nodes (8 nodes which includes V1, V2d, V2v, V3, V3a, V4, VP and V5 areas in each hemisphere) within 5-mm radius around the coordinates as ROIs in the visual streams to compute ROI-wise correlation matrix^39^. The coordinates of these nodes were selected and obtained from a previous study^40^. We also defined V1(Brodmann Area 17) and V2(Brodmann Area 18) areas in both hemispheres as seed ROIs to compute voxel-wise FC maps (Table 6, Fig. 5). Pearson’s correlation analysis was performed between each pair of time courses in these 16 nodes and between the seed reference time course and the time series from the whole brain in a voxel. The correlation coefficients were converted to z values using Fisher’s r-to-z transformation.

**Table 6:** The coordinates of ROI-wise seed nodes.

Brain region	Peak MNI coordinate		
	x	y	z
V1(L)	−6	−87	2
V2v(L)	−7	−79	−5
V2d(L)	−11	−96	8
VP(L)	−14	−76	−8
V3(L)	−18	−93	12
V3a(L)	−26	−87	17
V4(L)	−22	−70	−8
V5(L)	−41	−75	3
V1(R)	8	−83	5
V2v(R)	10	−74	−1
V2d(R)	10	−91	11
VP(R)	15	−73	−6
V3(R)	15	−91	16
V3a(R)	20	−86	21
V4(R)	22	−77	−11
V5(R)	46	−66	−1

### Data Availability

The datasets generated and analysed during the current study are available from the corresponding author on reasonable requests.
